# Reclaiming Rural Medicine: Mass Drug Administration, Community Sovereignty, and Institutional Memory

**DOI:** 10.7759/cureus.99372

**Published:** 2025-12-16

**Authors:** Arya Babul, Parisa Mahdavi, Amna Hussain, Zaigham H Janjua, Momina Hussain

**Affiliations:** 1 Biomedical Sciences, West Career and Technical Academy, Society for Awareness of Neglected Diseases, Las Vegas, USA; 2 Drug Development, Tavolar LLC, Las Vegas, USA; 3 Pediatrics, Dr. Akbar Niazi Teaching Hospital, Islamabad, PAK; 4 General Medicine, Dr. Akbar Niazi Teaching Hospital, Islamabad, PAK; 5 Genomics, Chinese Academy of Tropical Agricultural Sciences, Sanya, CHN

**Keywords:** community sovereignty, epistemic exclusion, epistemic indifference, health equity, mass drug administration, neglected tropical diseases, preventive chemotherapy, rural medicine, workforce memory, world health organization

## Abstract

Rural medicine has generated pragmatic, scalable solutions, exemplified by mass drug administration (MDA) that advance disease control and social equity. This editorial argues that rural practice should be reclaimed as scientific capital by centering on three pillars: evidence‑driven MDA integrated with complementary interventions; workforce memory treated as infrastructure; and measurable community sovereignty metrics to ethically guide population‑level interventions. Drawing on historical and contemporary neglected tropical disease (NTD) programs, it outlines practical steps for implementation, recommends routine debriefs and recorded community histories, and proposes simple measures to assess whether local consent and governance are sufficient to act. It also calls on funders, academic institutions, and implementers to invest in rural learning sites, protect institutional memory, adopt community‑centered metrics, and consider emerging tools such as artificial intelligence (AI) to strengthen logistics and community governance. Far from being a deficit, rural medicine offers a reservoir of pragmatic knowledge directly relevant to underserved U.S. communities. MDA or preventive chemotherapy provides adaptable tools for rural settings through community engagement, flexible logistics, and local decision‑making. Drawing on practical, not exotic lessons from past and current NTD programs, the editorial argues that MDA‑informed, community‑led approaches could, at a minimum, inspire and potentially help reduce health disparities in Appalachia, the Mississippi Delta, the Navajo Nation, and among migrant farmworker populations. The editorial further highlights the vulnerability of institutional memory in the wake of funding cuts and considers how AI could reinforce logistics and embed community governance in rural health interventions.

## Editorial

It is about knowledge, about knowing and not knowing. It is about the ethical responsibility to be aware, to pay attention, to not look away, to have respectful regard for the lives of other and othered people, to affirm their dignity as knowers, to attend to their knowledge, sensemaking, experiences, and aspirations, on their own terms. It is about how indifference breeds epistemic injustice.

 ­­Seye Abimbola (2025)

Introduction

“Mshamba.” “Bush league.” “The sticks.” “土包子” (tǔbāozi). Across languages and cultures, including Swahili, English, and Mandarin, rural communities are routinely caricatured as backward, unsophisticated, or out of step with modern medicine. These pejorative terms reflect a persistent urban bias that is, at best, epistemic indifference and, at worst, epistemic exclusion: the presumption that knowledge, rigor, and innovation reside elsewhere.

Neglected tropical diseases and mass drug administration

Some of the most resilient, innovative, and impactful health interventions have emerged in so‑called peripheral settings. In tropical regions, rural medicine is not a footnote; it is frontline practice. One example is mass drug administration (MDA), the periodic treatment of at‑risk populations without prior individual diagnosis, also known as preventive chemotherapy. MDA targets neglected tropical diseases (NTDs), a diverse group of debilitating bacterial, parasitic, viral, and fungal infections endemic to low‑resource regions of Africa, Asia, and South America. It often enables simultaneous, cost‑effective treatment of co‑endemic NTDs delivered annually or biannually [1-3].

**Figure 1:**
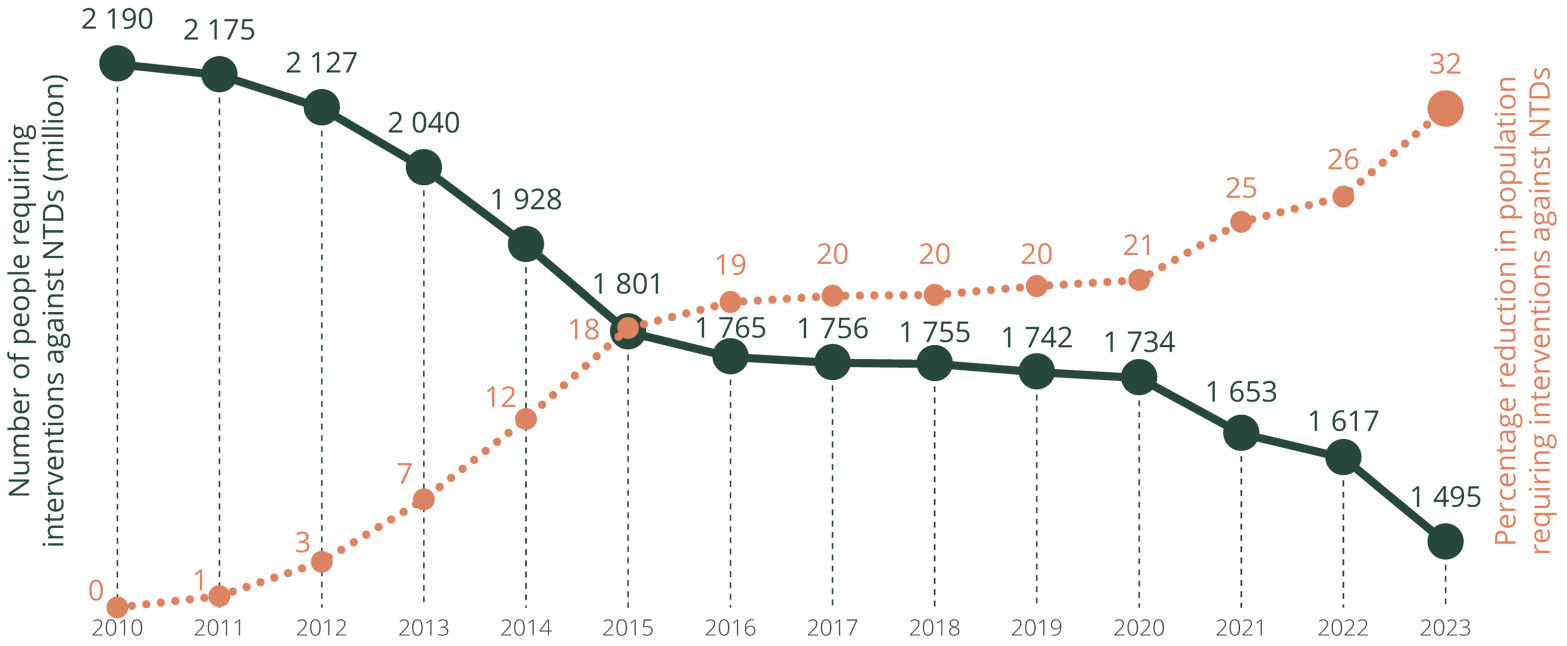
Number of people requiring interventions against NTDs in 2010–2023 and percentage reduction since 2010. Data source: WHO/Global Health Observatory. Reproduced from global report on neglected tropical diseases 2025. Geneva: World Health Organization; 2025 [2]. Creative Commons Attribution-NonCommercial-ShareAlike 3.0 IGO license: CC BY-NC-SA 3.0 IGO. WHO/Global Health Observatory. NTD = neglected tropical disease; WHO = World Health Organization

Currently, the World Health Organization (WHO) identifies 21 major conditions as NTDs, which disproportionately affect approximately 10% of the global population, primarily indigenous communities living in extreme poverty [1,2]. In 2023, an estimated 1.495 billion people required interventions for NTDs, with approximately 1.493 billion (99.86%) eligible for MDA. Of these, 865 million were treated for at least one NTD. From 2010 to 2023, the number of people requiring interventions decreased by a staggering 32%, from 2.190 billion to 1.495 billion, reflecting the combined efforts of the WHO, the now‑defunct United States Agency for International Development (USAID), philanthropic organizations, global non-governmental organizations, and national initiatives (Figure 1). MDA provides low-cost interventions capable of transforming entire regions by interrupting the poverty cycle linked to chronic illness, reduced productivity, impaired cognitive development, and social stigma [1,2]. Such programs require epidemiological rigor and dependable community trust, qualities honed by rural healthcare providers and field researchers.

The introduction of MDA was not merely a semantic innovation; it represented a strategic reorientation with profound implications for policy, practice, stigma, and outcomes. Early examples include the Rockefeller Sanitary Commission’s public health campaign in the southern United States (1909-1914), which combined mass treatment with sanitation, infrastructure, and public education, yielding measurable gains in school attendance and economic productivity [4]. Its success laid the foundation for modern public health systems and later informed WHO-led programs and contemporary MDA platforms [2].

Although interventions such as clean water, improved sanitation, hygiene promotion, routine immunization, and antibiotics remain cornerstones of global health, MDA has proven to be a humane, scalable, and often cost‑effective component of integrated control strategies for many NTDs. Cost‑effectiveness and program‑evaluation studies of MDA‑based approaches demonstrate important population benefits when delivered with strong monitoring, community engagement, and resistance surveillance [5].

Debates in high‑income settings have emphasized individual autonomy, informed consent, and the potential harms of non‑selective drug delivery (i.e., treating entire populations regardless of infection status) [6]. Those debates are necessary, but when framed abstractly, they can obscure important tradeoffs: untreated reservoirs of infection sustain transmission, and population‑level interventions can deliver large health and socioeconomic gains. NTDs operate within tangled ecological webs involving humans, livestock, wildlife, insect vectors, and environmental reservoirs. Transmission is cyclical, seasonal, and often invisible. Unlike chronic conditions that primarily respond to individual medical management, controlling NTDs is less like a solo and more like conducting an orchestra: public health, sanitation, vector control, supply chains, and community leadership must play in concert.

Reframing ethics for population-level rural interventions, such as MDA or preventive chemotherapy, means moving past a binary of individual autonomy versus aggregate benefit toward a pragmatic framework grounded in community sovereignty metrics, i.e., indicators that capture community control over decisions, resources, and program continuity. In the United States, such a framework must be adapted to existing legal and regulatory norms. This includes embedding informed consent into community-approved protocols, clarifying liability protections through public health statutes, and piloting programs under local health departments rather than private payers. In practice, this could mean staged approvals in low‑income rural towns where local health boards authorize MDA campaigns, paired with opt‑out provisions and independent oversight to ensure compliance with U.S. legal and regulatory norms. Ethical pragmatism does not abandon consent or individual rights; rather, it embeds those rights within measurable structures of local governance, shared decision‑making, and accountable implementation so population strategies such as MDA are simultaneously effective and ethically defensible.

By making these dimensions measurable, programs can convert abstract ethical arguments into concrete performance targets that stakeholders must meet before and during population interventions. Practically, this means using staged approval criteria: a routine MDA can proceed once core sovereignty metrics are met (for example, a community‑approved communication plan), while higher‑risk or more intrusive actions require stronger, wider support from different groups inside the community.

Embedding simple measures of community control into regular monitoring changes how we think about autonomy: it stops being a check‑box for each person and becomes a shared, improvable feature of how projects are run. Ethically, this keeps room for individuals to refuse while also creating community consent processes that have real local legitimacy. For those weighing harms and benefits, these measures make the trade‑offs tangible. In short, using community control as a practical yardstick turns abstract ethical considerations into concrete design choices that align scientific goals with democratic accountability and restore the community’s voice in how knowledge and decisions are made.

In 2024, the WHO reported that seven countries had eliminated an NTD, bringing the cumulative total to 54 countries with at least one eliminated NTD [2]. Nonetheless, MDA programs face persistent challenges. A recent Cochrane review [3] found a mix of physical and social barriers that keep willing people from accessing treatment: historical grievances, rumors, and mistrust reduce confidence in drug distribution; drug distributors often hold low status in their communities and lack the training to answer questions; many distributors have limited clinical background; community members sometimes do not know when or why distributions occur; residents want greater local involvement in program design; and demand for complementary measures (for example, improved sanitation) is high. Additionally, WHO recommends MDA against five of 21 NTDs, namely, lymphatic filariasis, onchocerciasis, schistosomiasis, trachoma, and soil-transmitted helminthiases; however, up to about a dozen NTDs are amenable to MDA [2].

Institutional memory as the backbone of knowledge continuity

The United States has been influential in the development of tropical medicine through USAID, the Centers for Disease Control and Prevention, and the National Institutes of Health, not only via field stations, collaborative trials, and partnerships but also through sustained financial support to the WHO and its NTD initiatives. However, recent shifts in funding priorities and workforce stability, including the dismissal of thousands of public health experts, have weakened elements of that infrastructure. While philanthropy and private entities are filling some gaps, they cannot entirely replace sustained public investment and institutional memory [7,8].

For example, Johns Hopkins University, a global center of excellence in tropical medicine and infectious disease research, has laid off more than 2,200 employees worldwide, including 1,975 employees across 44 countries, 247 in the United States, and furloughed additional staff after the abrupt termination of $800 million in USAID funding [8]. Similar cuts triggered mass furloughs at RTI International, a large non-profit research institute, and FHI 360, a global non-profit working on health and development programs, affecting hundreds of staff across North Carolina and other global health hubs [9]. Many of these grants supported programs targeting tropical diseases, maternal care, and clean water access, and their loss has forced the closure of dozens of field sites and disrupted hundreds of clinical trials across Africa, Asia, and Latin America. Further reductions in both enacted and proposed NIH funding, along with ongoing uncertainty, have compounded these impacts, straining research continuity and domestic staffing across multiple academic and non-profit institutions. A report published in November 2025 by Patel and colleagues in JAMA Internal Medicine found that disruptions affected roughly 1 in 30 NIH‑funded clinical trials, involving more than 74,000 participants [10]. Trials conducted outside the United States were disproportionately affected (5.8% vs. 3.4% for U.S.‑based trials), with the greatest losses seen in studies of infectious diseases, prevention, and behavioral interventions.

Employee turnover and attrition can occur routinely, but the current high-profile and large‑scale departures underscore a clear vulnerability: when experienced field professionals and local stakeholders depart, programs lose more than people; they lose operational bandwidth, practical records, know‑how, and informal problem‑solving practices that kept programs running in challenging environments. That loss of institutional memory weakens the infrastructure of evidence and experience.

Importantly, the erosion of institutional memory is not simply an occurrence in under‑resourced regions with technological or literacy limitations. Even in well‑resourced settings, such as pharmaceutical and biotech companies, mergers and downsizing have led to the loss of translational medicine expertise and hard‑won lessons in biomarker development [11]. Similarly, academic medical centers have experienced diminished continuity when senior leaders retire or programs reorganize, weakening the transmission of clinical knowledge and institutional history [12]. These examples highlight that institutional memory is universally vulnerable, and its preservation requires deliberate structures regardless of geography or resource level.

Treating workforce memory as infrastructure means valuing routine practices and taking deliberate steps to protect, capture, and share them. Practically, this means using standard debrief forms, recording short oral histories with community partners, and keeping concise “how we did it” guides. To strengthen feasibility under unstable funding conditions, such memory‑capture mechanisms must be designed to survive abrupt investment shifts. Independent, low‑cost repositories, such as open‑access digital archives or community‑based knowledge banks supported by professional associations or local health boards, can safeguard oral histories and debriefs even when institutional staffing is disrupted [11]. For example, state public health associations could host open‑access archives that preserve debrief forms and oral histories, ensuring continuity of tacit knowledge even when federal funding shifts disrupt local staffing. Such repositories would provide a durable, low‑cost mechanism for capturing institutional memory outside the lifecycle of individual grants. These structures provide continuity of tacit knowledge beyond the lifecycle of individual grants.

Beyond preservation, workforce memory can accelerate scale‑up and strengthen resilience by supporting repeatable training, mentorship, and a redeployable pool of implementers. When captured knowledge is paired with simple metrics, it becomes auditable and improvable, turning “remembered craft” into measurable assets. Ethically, prioritizing workforce memory addresses an epistemic injustice by recognizing the expertise of field staff and community health workers as scientific capital rather than disposable labor. In short, treating workforce memory as both a vulnerability and an asset reframes layoffs and site closures from irreversible losses into recoverable, shareable institutional knowledge.

Pragmatic implementation science for rural health systems

Rural health settings are ideal for adaptive implementation because they force practical solutions when standard protocols break down. Treating clinics, community health workers, and local campaigns as learning sites turns constraints into advantages: small teams can test quick changes, track simple indicators, and decide locally whether to adopt, adapt, or stop an approach. Such learning yields improved local fit and practical, transferable strategies, for example, how to coordinate water and drug interventions to increase coverage. Over time, those local experiments create a library of practical strategies that policymakers can scale as adaptable logic rather than rigid programs.

Looking ahead, artificial intelligence (AI) offers another potential lever for rural health equity. Building on Volandes and colleagues’ November 2025 perspective in the New England Journal of Medicine, AI is increasingly being positioned as a tool for transforming rural care delivery [13]. For AI to synergize with and not disrupt rural health systems, it must be integrated into the social support networks that sustain patients in their homes and communities. That means systems should provide clear, easy‑to‑act‑on information, respect local caregiving practices and limited infrastructure, and link digital alerts to people who can follow up. Just as many rural communities in sub‑Saharan Africa and other underserved regions worldwide skipped landlines and went straight to mobile phones, rural health systems today may be able to “leapfrog” traditional bottlenecks by using AI. Applied to MDA and NTD programs, such AI‑enabled tools could improve logistics and bring community oversight into digital platforms. Far from replacing human relationships, such technologies could extend the reach of rural wisdom, making population‑level interventions both more effective and more accountable.

Limitations and implications

Despite the promise of MDA and community‑led rural strategies, important limitations warrant acknowledgement. MDA can produce unintended harms, including adverse drug reactions, the potential for accelerating antimicrobial or anthelmintic resistance, and the risk of coercive practices when community engagement is superficial. Program effectiveness also depends on reliable supply chains, surveillance systems, and complementary investments in water, sanitation, and primary care, resources that are often scarce in the same settings MDA targets. Evidence gaps remain about long‑term socioeconomic benefits and about how best to adapt MDA to populations with high seasonal mobility or complex comorbidities [5,6]. To mitigate these risks, programs must pair sovereignty metrics with robust pharmacovigilance, resistance monitoring, and independent evaluation; require transparent reporting of harms as well as benefits; and treat MDA as one component of integrated, locally governed health strategies rather than a standalone solution.

Conclusions

Far from being a deficit, rural medicine offers a reservoir of pragmatic knowledge whose lessons abroad are directly relevant to underserved communities in the United States and other advanced economies. MDA provides practical tools that can be adapted to rural settings through community engagement, flexible logistics, and local decision-making. To translate these lessons into policy, future efforts should preserve workforce memory through resilient repositories, where indicated, pilot ethically safeguarded MDA programs, and embed measurable community governance into funding and implementation. Taken together, these steps reclaim rural medicine as scientific capital and ensure equity is built into population-level health interventions. The future of medicine may well depend on places we have long ignored and people we have long underestimated.
